# Anatomic Revascularization of the Celiac Trunk and the Superior Mesenteric Artery

**DOI:** 10.1055/s-0038-1639378

**Published:** 2018-07-27

**Authors:** Murat Ugurlucan, Nihat Aksakal, Yilmaz Onal, Didem Melis Oztas, Ufuk Alpagut

**Affiliations:** 1 Department of Cardiovascular Surgery, Istanbul University Istanbul Medical Faculty, Istanbul, Turkey; 2 Department of General Surgery, Istanbul University Istanbul Medical Faculty, Istanbul, Turkey; 3 Department of Radiology, Istanbul Sultan Abdulhamid Han Training and Research Hospital, Istanbul, Turkey

**Keywords:** atherosclerosis, mesenteric ischemia, surgical revascularization

## Abstract

Chronic atherosclerotic mesenteric ischemia is a debilitating disorder. It may cause postprandial abdominal pain leading to severe weight loss. Patients are usually emotionally affected with major depression. The disease can be treated with open surgical and endovascular techniques and both methods have individual risks and benefits. In this report, the authors present anatomical revascularization of the superior mesenteric artery and the celiac trunk.

Chronic mesenteric ischemia is a rare condition, which results from decreased intestinal blood flow due to atherosclerosis in at least two of the splanchnic arteries, that is, the celiac trunk, superior mesenteric artery, or the inferior mesenteric artery, which are interconnected via multiple collaterals. 1 It is an important cause of postprandial abdominal pain that requires treatment, otherwise, this may lead to severe malnutrition or intestinal gangrene, and even to death. 1 2

A 47-year-old male patient was referred to our institution with the diagnosis of occlusion of the celiac trunk and the superior mesenteric artery ( Fig. 1 ). He lost 20 kg in 3 months because of inability and fear of eating due to severe abdominal pain after meals. He was an ex-smoker who otherwise did not have any hereditary or acquired risk factors for atherosclerosis. He underwent descending aorta to celiac trunk and superior mesenteric artery bypass grafting with a Y-saphenous vein graft through median laparotomy ( Fig. 2 ). The duration of the operation, intensive care unit, and hospital stays were 6 hours, 24 hours, and 8 days, respectively. His symptoms relieved immediately following surgery and he started to gain weight. He has been followed uneventful in good health status for more than 18 months.

**Fig. 1:**
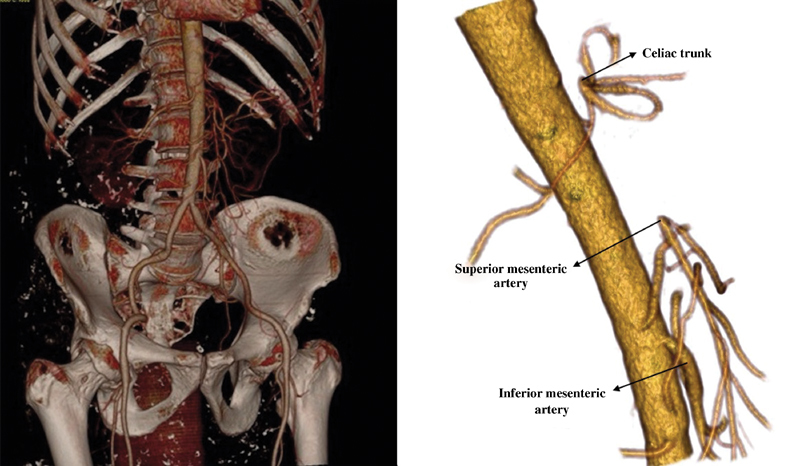
Computed tomography angiography showing occluded celiac trunk and the superior mesenteric artery.

**Fig. 2:**
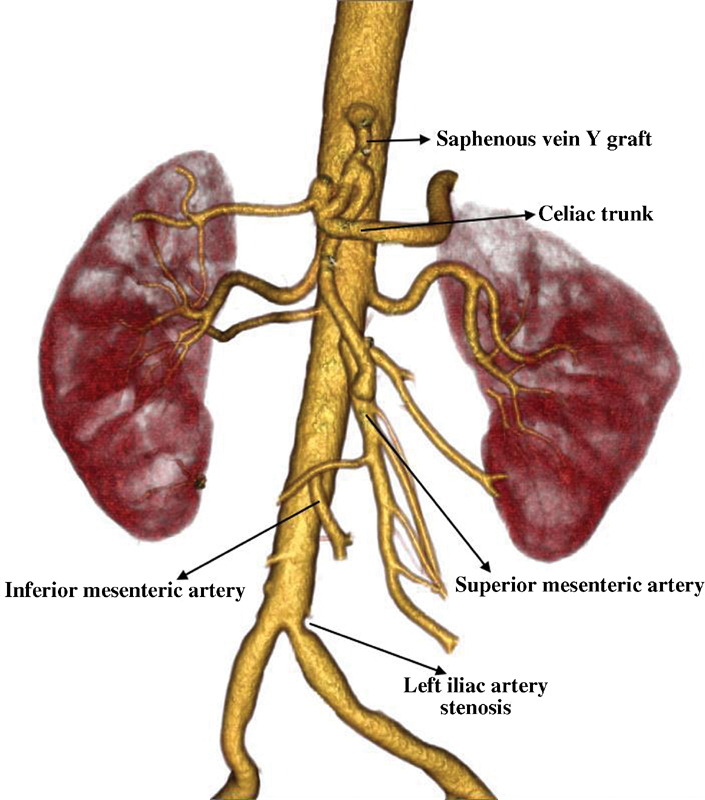
Control computed tomography angiography showing the aortoceliac trunk and superior mesenteric artery bypass with Y-shaped saphenous vein graft and minimally stenosed left common iliac artery.

Surgical or interventional treatment is indicated in symptomatic patients with mesenteric ischemia. Open surgical revascularization is associated with immediate relief of symptoms and long-term durability. It is also associated with certain mortality and morbidity rates. In addition, the complex anatomy and difficult exposure of the splanchnic area have been another drawback of conventional surgical revascularization. Percutaneous endovascular techniques may also be performed for mesenteric revascularization in selected cases. 1 2

When surgical treatment is aimed, complete anatomic revascularization from the aorta to the affected arteries should be the aim; however, extra-anatomic retrograde bypass from one of the iliac arteries may also be performed for the ease of the technique. 3 Endovascular revascularization was not attempted due to long segment occlusion of the celiac trunk and the superior mesenteric artery in our particular case. Young age of the patient was the other reason for the preference of surgical revascularization of the affected arteries.

We preferred the descending aorta as the inflow artery. It has not been difficult to reach the descending aorta through median laparotomy due to the very thin posture of the patient. However, it may be challenging in obese cases. In our patient, the bilateral iliac arteries were affected with atherosclerosis with the left iliac artery containing 30% stenosis which was omitted to interfere. It was another reason to opt out of the iliac arteries as an inflow vessel.

We believe that our anatomic splanchnic revascularization with the use of an autologous graft material has been an effective treatment option for our particular case.

## References

[1] Nazlı Y Çolak N Şahin H Surgical treatment of chronic mesenteric ischemia with splenic artery-to-superior mesenteric artery bypass: a case report Turk Gogus Kalp Dama 2013 21 02 463 466

[2] Kasirajan K O'Hara P J Gray B H Chronic mesenteric ischemia: open surgery versus percutaneous angioplasty and stenting J Vasc Surg 2001 33 01 63 71 11137925 10.1067/mva.2001.111808

[3] Christopoulos D Podas T Pitoulias G Tachtsi M Papadimitriou D S-shaped ilio-mesenteric bypass in a young high risk patient Int Angiol 2008 27 04 353 355 18677300

